# Empowering Chinese university health service providers to become mental health champions: insights from the ACE-LYNX intervention

**DOI:** 10.3389/fpsyt.2024.1349476

**Published:** 2024-03-22

**Authors:** Fenghua Wang, Jianguo Gao, Suyu Hao, Ka Tat Tsang, Josephine Pui-Hing Wong, Kenneth Fung, Alan Tai-Wai Li, Cunxian Jia, Shengli Cheng

**Affiliations:** ^1^ Department of Social Work, School of Philosophy and Social Development, Shandong University, Jinan, Shandong, China; ^2^ Department of Social Work, Law School, Qingdao University of Science and Technology, Qingdao, Shandong, China; ^3^ Factor-Inwentash Faculty of Social Work, University of Toronto, Toronto, ON, Canada; ^4^ Daphne Cockwell School of Nursing, Toronto Metropolitan University, Toronto, ON, Canada; ^5^ Department of Psychiatry, University of Toronto, Toronto, ON, Canada; ^6^ Primary Care, Regent Park Community Health Centre, Toronto, ON, Canada; ^7^ Department of Epidemiology, School of Public Health, Shandong University, Jinan, Shandong, China

**Keywords:** empowerment, Chinese university students, mental health, service providers, mental health champions, empowerment psychoeducation

## Abstract

**Background:**

Evidence shows that there is a high prevalence of mental health challenges including depression and anxiety, among Chinese university students. Providing mental health care providers with professional training is crucial to implementing effective mental health promotion initiatives in university settings. Globally, the focus of the mental health system is shifting to recovery and the importance of empowerment is increasingly being emphasized. There is a call to integrate empowerment education into professional training programs for health service providers with the goal of mobilizing them to become mental health champions capable of advancing mental health care.

**Method:**

The ACE-LYNX (Acceptance and Commitment to Empowerment–Linking Youth and Xin i.e., “heart(s)” in Chinese) intervention took place at six universities in Jinan, Shandong Province, China. It aimed to promote mental health literacy and build capacity among mental health service providers (MHSPs) to enable them to become mental health champions at their universities and beyond. A total of 139 university MHSPs participated. We collected pre-, immediate post- and three-month-post-surveys. In addition, we recruited forty-five participants to take part in three-month- post-intervention focus group interviews to explore their experiences taking part in ACE-LYNX and applying the knowledge, skills, and insights they gained from the intervention.

**Result:**

This paper reports on the effects of empowerment education, which is a key component of ACE-LYNX, on the MHSPs. Four themes were identified: 1) conscious awareness and behavioral change through psychological empowerment users; 2) professional insights and motivation for organizational empowerment; 3) non-self in the continuum of collective empowerment; and 4) interdisciplinary challenges and divergences in empowerment action.

**Discussion:**

We found that it is critical to integrate empowerment education into professional training. The process of MHSPs developing their empowerment practice is characterized by their moving from individual to collective empowerment along a continuum, with organizational and collective empowerment taking place in a longer time frame. Experiential learning, empathy education, and critical reflection accelerated the continuous iterative transformative process of empowerment practices. To advance the integration of empowerment into mental health care, the engagement of organizational decision-makers and policy makers in empowerment training is critical to ensure alignment of empowerment values and competence at all levels of service provision.

## 1 Introduction

### 1.1 Mental health among Chinese university students

The overall prevalence of various mental disorders among Chinese university students remains is high. About 20–45 percent of Chinese university students are at risk of depression and anxiety (1), and sleep problems, somatic problems, and self-injury are also prominent (2, 3). Evidence suggests that there is a trend toward gradual deterioration in student mental health after experiencing COVID-19 (4). However, many students do not seek help when they are faced with mental health challenges, and find themselves in need of psychological treatment and support. In addition to the stigma associated with mental illness (5) and low mental health literacy (6), the lack of adequately trained mental health service providers (MHSPs) in university settings is a hindrance (7). Currently, psychosocial interventions for Chinese university students are mostly delivered by students affairs counselors and teachers, family members, health care authorities and psychological counselors (8), who are mostly non-mental health professionals. To advance mental health care for university students, there is a need to build the capacity of existing service providers to respond effectively to students’ mental health needs. Policymakers and researchers recommend strengthening service teams and networks in universities (9, 10) and carrying out more health promotion measures and interventions to help students access better mental health care (11).

### 1.2 Empowerment in mental health promotion

In mental health service systems, empowerment is increasingly identified and embraced as a desired overall health outcome (12). In 2010, the World Health Organization advocated for making the empowerment of mental health service users as a health system priority (13). Subsequently, governments and policymakers in many countries have integrated empowerment into their frameworks guiding the provision of mental health services (14–19). As indicated in the following sections, the middle range theory of empowerment has long been applied in the fields of community psychology, community development, and health promotion in general. The 1986 Ottawa Charter for Health Promotion (20) defines health promotion as “the process of enabling people to increase control over, and to improve, their health … At the heart of this process is the empowerment of communities, their ownership and control of their own endeavors and destinies” (p. 3). Thus, empowerment can be defined in terms of both processes and outcomes operating at the individual, organizational, and community levels (21).

Psychological empowerment is a term commonly used to describe empowerment at the individual level, taking into account the reciprocal influences of individuals on society and of social forces on individuals (22). Zimmerman further identifies three key aspects of psychological empowerment: 1) intrapersonal – an individual’s sense of self-efficacy, belonging, motivation, and competence; 2) interactional – an individual’s critical understanding of what affects their health and well-being and their knowledge of how to access resources in order to gain control of their health; and 3) behavioral – individuals taking action to improve their health and well-being (23). Paulo Freire suggests that critical thinking emerges when individuals gather to engage in dialogue and reflection on the social determinants of their lived experiences and well-being (24). In this sense, psychological empowerment occurs when individuals gather to identify and discuss their health challenges and collaborate to find solutions (25).

Individuals are able to connect, engage in dialogue, and collaborate to find solutions through organizations. At the same time, an organization is built on and sustained by the strengths of positive relationships among its members. An organization becomes empowered when it is able to build partnerships and mobilize its members and community stakeholders effectively to work towards improving the health and wellbeing of the communities it serves (26, 27). Thus, it can be seen that psychological empowerment and organizational empowerment are intertwined and function together as the foundation of community empowerment, which can be understood as a “social action process, that promotes participation of people, organizations, and communities towards the goals of increased individual and community control, political efficacy, improved quality of community life, and social justice.” (28).

Evidence indicates that service users have a desire to feel empowered in accessing mental health care (29). Similarly, how to foster a sense of empowerment has been a focus of research on university students’ mental health (30). There has been a considerable amount of research on mental health in empowerment interventions for people with various psychological problems (31–33), but most studies have only focused on the outcomes for service users. There is little research on how to promote the psychological empowerment of MHSPs as a intertwined strategy to promote empowerment as a core competency in delivering effective mental health care (34). As the effectiveness of strength-based empowerment practices has been demonstrated, empowerment training for MHSPs is needed to promote their professional capacity and core competence (35). In addition, evaluative research is needed to examine the processes and outcomes of empowerment education for MHSPs to inform the design and implementation of such programs (36, 37). Since empowerment consists of complex processes involving many stakeholders (e.g., service users, MHSPs, administrators, advocates, etc.) who are involved in relationship building, social interaction, and collaboration with each other, the inclusion of MHSPs’ subjective experiences, perspectives, and empowerment as a key component of program evaluation is very important. Research on the experiences and post-training action of MHSPs will generate knowledge to inform innovative strategies to increase empowerment practices among MHSPs (38). In this paper, we report on the results of our focus groups with MHSPs who completed an intervention that included collective empowerment as a key component.

### 1.3 The ACE-LYNX intervention

Acceptance and Commitment to Empowerment–Linking Youth and *“Xin”* (Chinese for “heart(s)”) (ACE-LYNX) is an evidence-based intervention designed to build capacity among MHSPs in mental health literacy and strength-based services. ACE-LYNX is a key component of the Linking Hearts Project, an international, multidisciplinary, collaborative implementation science study conducted between Canada and China. The learning activities in ACE-LYNX have been evaluated and found to be effective (35–37). Prior to the adoption of ACE-LYNX for use in China, we undertook a contextual assessment and analysis of 600 university students and 144 MHSPs to guide the localization of the intervention (39). The results from the contextual assessment were used to guide the local adaptation of the evidence-based intervention into ACE-LYNX. The overall goal of ACE-LYNX is to increase the capacity of MHSPs working in university settings to become mental health champions and to provide strength-based empowering mental health care. Furthermore, these multidisciplinary champions are supported to develop formal and informal mental health networks and engage in collaborative care (39). In addition, they are involved in knowledge translation and dissemination.

ACE-LYNX is an integrative intervention based on the Acceptance, and Commitment to Empowerment (ACE) model (38). The ACE model transforms two well-established mental health promotion models into one comprehensive model. It is an integration of (1) Acceptance and Commitment Therapy (ACT) (40) and (2) Group Empowerment Psychoeducation (GEP) (41–43). ACT is a mindfulness-based cognitive behavioral intervention that promotes psychological flexibility and supports psychological empowerment. It consists of six processes: “defusion (observing thoughts as thoughts), acceptance (opening up to experiencing thoughts and feelings), contact with the present moment (attending to the present mindfully), self-as-context (being in touch with the ‘observer self’ and increasing perspective-taking skills), values (being clear about what matters), and committed action (developing consistent patterns of behaviors based on one’s chosen values)”. In ACE-LYNX, the GEP component is underpinned by the principles and values of social justice and equity, as well as empathy and compassion. The training emphasizes interdependence among people and promotes collective empowerment through four processes: critical reflection, critical dialogue, collaborative learning, and experiential learning (38). ACE-LYNX is unique in that the ACT and GEP concepts are fully integrated and operationalized to promote both psychological and collective empowerment.

### 1.4 Intervention process

The integrated ACE-LYNX intervention was operationalized and delivered via two complementary modalities (1): an online self-study course named Mental Health 101(MH101) and (2) a five-day experiential group training. The MH101 module addresses the following knowledge elements: concepts of mental health and well-being; factors that adversely affect mental health; common mental disorders; and resources for disease prevention, treatment, and recovery. The five-day ACE-LYNX in-person training (**Table 1**) engaged MHSPs in three days of experiential collaborative learning and two days of practicing the newly gained ACE-LYNX skills to prepare them to take on the role of mental health champions and transfer the knowledge and skills gained in the program to their colleagues, students, service users, and others. Since the purposes of the ACE-LYNX training were to promote interdisciplinary collaboration and expand on-campus capacity to provide mental health care to university students, the participants included medical and mental health professionals, as well as non-health professionals such as faculty/educators and student affairs advisors. The design and interactive delivery of the program drew on the principles of experiential and collaborative learning theory were appropriate for adult learning. Face-to-face group learning was organized with 20–30 participants per group. ACE-LYNX was designed to apply a mixture of different learning strategies: cognitive (e.g., theoretical concepts of the ACE model), kinesthetic (e.g., the “chair sculpture of suffering” exercise), audio-visual (e.g., paired singing), solitary (e.g., the leaves-on-a-stream” mindfulness exercise), and interactive (e.g., the “your legacy” exercise). All of these learning activities included group discussion and debriefing in the context of psychological and collective empowerment to encourage critical dialogue and reflection among the participants. The project was carried out in three phases and applied a “train-the-trainer” model (44), whereby the trained MHSPs became champions who directly implemented empowerment education with the opportunity to implement ACE-LYNX and empowerment education with groups of MHSPs and students in subsequent phases of the project. The champions received printed copies of the ACE-LYNX learning materials, including the MH101 course materials, evidence-based research papers on ACE-LYNX, and the ACE-LYNX training manual. All of the newly trained champions joined a three-month *Community of Practice* practicum, during which they kept connected with their trainers and peers to learn from each other. They were also invited to complete monthly activity logs to capture their regular reflections on the impact of the intervention on their personal and professional lives. Additionally, these champions had access to and participated in ongoing supervision, digital capacity building meetings with Canadian team members, local support through internet communication spaces (WeChat discussion groups), and the annual team seminar. The Community of Practice was designed to not only build mental health care competence (45, 46) among MHSPs but also structure peer support, relationships building and collaborative learning (47), which are key to both individual and collective empowerment.

**Table 1:** ACE-LYNX training.

Time		ACE Concepts & Processes	Learning Activities
DAY 1	AM	Group Values	Introduction to ACE theory; Building Group Norms
	PM	Present Moment; Defusion; Empathy & Compassion	Present moment I; Leaves-on-a-stream; Paired singing, and stigma rules and stories
DAY 2	AM	Defusion; Acceptance, Values; Social justice & Equity; Collective empowerment	Chair sculpture of suffering; Exclusion circle
	PM	Self-as-context; Defusion, Compassion, Values, Interdependence	Le’go exercise; Three things you did today
DAY 3	AM	Values; Interdependence	Cultural and personal values exercise; Your legacy
	PM	Committed action, Value, collective empowerment	Bulls-eye; Bus driver; Collective bulls-eye and commitment group action project
DAY 4	AM	Reflection and feedback	Discussion and share
	PM	ACE model review and facilitation skills	Facilitating and leading ACE, and experiential learning, group exercises
DAY 5	AM	Mock group practice	Paired singing and chair sculpture of suffering
	PM	Mock group practice	Exclusion circle and bus driver
		Collaboration: A functional MH network	Group discussion

## 2 Methods

As an implementation science study, the Linking Hearts Project explored the effectiveness and characteristics of the ACE-LYNX, an empowerment education intervention as a strategy for building the capacity of MHSPs working with university students to provide strength-based and empowering mental health services. In this paper, we focus on the MHSPs’ knowledge, attitudes and competence in empowerment practices after completing the ACE-LYNX training, paying special attention to their critical reflections on and actions in providing mental health care to university students. We formulated two specific evaluation questions: 1) what were the MHSPs participants’ cognitive, behavioral, and affective responses to the ACE-LYNX intervention? 2) What are the characteristics of psychological and collective empowerment among the MHSP participants? These questions will enable us to generate insights and knowledge to better support MHSPs in their efforts to become the mental health champions and offer empowerment services in the university setting and beyond.

### 2.1 Participants in the ACE-LYNX intervention

Mental health services for students in Chinese colleges and universities are provided by a variety of MHSPs, including medical and mental health professionals, and non-health professionals such as faculty/educators and student affair advisors, who collaboratively work in the four-tier “university-school-class-dormitory” system, whereby the university provides policy directions that are integrated into every school and department and implemented by faculty and educators in the classroom and by student affairs advisors in the dormitory (9). We recruited139 MHSPs from six universities and a mental health care center in Jinan to participated in ACE-LYNX. In addition to the pre- and post-training questionnaires, 30 percent of the participants (n=45) were randomly selected and invited to participate in the post-training focus group interviews. The participants included five types of personnel: university advisors^1^, university teachers, psychological counselors^2^, mental health medical personnel^3^ and others (e.g., social workers) (see **Table 2**). The university advisors only had basic training in knowledge and skills related to mental health education, the psychological counsellors had degrees in psychology, and university teachers were predominantly social work faculty. Although the mental health medical personnel had received extensive biomedical training in psychiatry, their access to integrated training on psychotherapeutic or empowerment strategies was limited. Their participation in ACE-LYNX reinforced the project’s goal of fostering collaborative efforts to promote student mental health and provide comprehensive care. All of the participants signed an informed consent form. Of the forty-five people interviewed, seventeen were university advisors, seven were university psychology counselors, fourteen were social work teachers, and seven were mental health institution professionals.

**Table 2:** Occupation of participants of ACE-LYNX and Focus Group Interviewees.

Occupation	All participants(N=139)		Interviewees (N=45)	
	N	%	N	%
University advisors	91	65.4	17	37.8
University teachers	19	13.7	15	33.3
Staff from mental health care centers	15	10.8	7	15.6
University psychological counselor	11	7.9	6	13.3
Others (e.g., social workers)	3	2.2	0	0

### 2.2 Data collection

We conducted seven mixed focus groups; each group consisted of six to eight participants, and the focus groups ranged in length from 90 to 120 minutes. Three of the focus groups were face-to-face interviews, and due to the COVID-19 public health requirements, four were conducted online using the Tencent Meetings app. Each focus group was conducted by two facilitators and one note-taker, who were both project staff members. None of the ACE-LYNX researchers/trainers were present at the focus groups to ensure that the participants could share their thoughts about their experiences freely. There was no recognizable relationship between the research team and the participants.

In the focus groups, we used semi-structured interviews to explore MHSP participants’ experiences of engaging in empowerment education and their follow-up actions during the three months after completing the training. Our questions included the following: “What was your overall experience of participating in this program? How have you used what you learned from the program in your personal live and professional practice, especially in working with students? What was your experience in participating in activities to reduce the stigma of mental illness and promote mental health? The Interviews were conducted from November 2020 to October 2021. Each participant received a small honorarium as a token of appreciation, as per our research ethics protocol.

### 2.3 Analytical framework

As described earlier, the Acceptance Commitment and Empowerment (ACE) model (41) used in ACE-LYNX promotes psychological empowerment through six psychological processes. Evidence shows that when individuals develop more psychological flexibility, they are better able to experience psychological empowerment in terms of self-efficacy, positive self-concepts, and self-compassion, which function together to increase their empathy for self and others, and reduce the psychological barriers to taking committed action for health equity (36, 37). In addition, the four pedagogical processes in the ACE model work in tandem with the psychological processes to mobilize participants for conscious awareness, exchange of ideas, development of relationships, production of shared knowledge, and achievement of collective goals (48).

While the effectiveness of the ACE model has been documented for stigma reduction (49, 50) and collective action (36, 37), the nuanced processes and outcomes of individual and collective empowerment in mental health promotion have not yet been reported in detail. In this paper, in addition to the ACE model, we draw on the empowerment continuum model, conceptualized by Jackson and colleagues (40) and adapted by other critical health promotion researchers (41, 42). The empowerment continuum model encompasses individual awareness and action orientation, social connections and mutual support, getting organized to achieve collective goals, participation and shared leadership, and collective action (51–53). These scholars argue that people can move along this continuum, achieving a level of empowerment from the individual to the organizational, and ultimately to the collective (community). By applying this model in our data analysis, we were able to illustrate the participants’ movement from psychological empowerment to collective community empowerment.

### 2.4 Data analysis

The audio recordings of the focus group were transcribed verbatim in Chinese, and the NVivo 13 software was used for data management. Thematic analyses were conducted by the three lead authors who belong to the Chinese team. The first author is a doctoral candidate and the third author is an early career doctorate in the discipline of social work. The second author is a senior research scientist specializing in social service and equity. The thematic analyses and interpretation were reviewed by the fifth author, a bilingual (English-Chinese) senior nursing and public health research scientist on the Canadian team who specializes in implementation science, mental health promotion, immigrant health, and health equity. She has extensive experience working with community-based mental health organizations serving Chinese and other East Asian immigrants in Canada. The manuscript draft was reviewed by other co-authors from the disciplines of cultural psychiatry, primary care, public health, and social work in Canada and China (see author affiliation). The diverse disciplinary backgrounds of Chinese and Canadian team members have strengthened the interpretation of the study data. The Chinese team members offered rich contextual information on mental health care in Chinese universities, while the Canadian team members offered cross-cultural interpretation of these contexts. We conducted both deductive analysis guided by the ACE model and empowerment theories, and inductive analysis informed by the voices of the participants (54). We applied the six steps described by Braun and Clarke to conduct a thematic analysis of the interview data (55, 56). First, to familiarize ourselves with the data and to find key ideas related to the subject of the study, the transcripts were read verbatim and line by line. Second, we formed an initial code list by combining the conceptual connotations of the different levels of empowerment, the empowerment concept, the learning methods in the ACE model, etc. Third, the clusters of similar codes were further grouped into potential themes and sub-themes (55). The first three authors reviewed, discussed, and refined the codes until a final set of codes was defined and named. They also exchanged and reviewed each other’s coded transcripts to reach agreement and enhance the trustworthiness and credibility of data analysis (57). They also engaged in reflexive dialogues with the fifth author (e.g., the different ways to interpret the participants’ narratives based on our position as students vs. a professor; cross-cultural and transnational interpretation of the concepts of empowerment, etc.) to promote rigor (58). To ensure the participants’ anonymity, all participants were identified by focus group number and participant number; for example, FG1-2 denotes Interviewee No. 2 in Focus Group 1.

## 3 Results

In this study, we identified four key themes about the empowerment process and its outcomes in our detailed analyses of the focus group interviews with the MHSPs. These four themes illustrate the participants’ cognitive, behavioral, and affective responses to empowerment practices. They began with self-awareness (59) and individual action to critically reflect on organizational constraints and possibilities for organizational empowerment (60), to recognize the interdependence of people and conditions for mental health, and to commit to collective empowerment. Their empowerment learning process demonstrated a continuum model of empowerment and was characterized by different paradigms of understanding and action in interprofessional teams.

### 3.1 Theme 1: conscious awareness and behavioral change through psychological empowerment

The MHSPs in the focus groups reported not only gaining psychological empowerment after participating in the ACE-LYNX training, but also the motivation to apply their new learning and insights in their interactions with students and other service users. They reconceptualized empowerment as practical wisdom they could apply in their daily mental health services for students.

First, many participants experienced an increase in their psychological flexibility, whereby they were able to go beyond misconceptions and stigma about mental illness. They reported that they continuously adjusted their attitudes toward students with mental problems and gradually moved beyond their original reactions of panic, distance, and rejection to respecting and supporting students with mental problems. These sharing illustrate the intrapersonal process of empowerment. They adopted a more open, accepting, and accommodating attitude to understanding the psychological conditions and complexities of the person’s life circumstances and engaged in actively listening, giving verbal encouragement and psychological counseling, and providing support.

*“Many of the students are in our school’s mental health tracking pool and are at risk for mental illness. I felt very headstrong and was tough with them at first. Since the training, my attitude toward them has shifted a lot, I’m not so scared. I think they have their own difficulties so I want to be more supportive of them, put more effort into them, and find a way to make sure that they are treated the same as normal classmates—equally or with even more care, I guess.”(FG2-3)*

Moreover, they changed their habit of attributing students’ psychological problems to individual weaknesses and paid more attention to the external objective and complex factors or events that triggered mental health problems. They expressed a greater understanding that psychological symptoms are the result of multiple factors rather than seeing them as signs of low personal resilience, poor ability, or psychological vulnerability. They were able to identify the complex hindrances faced by students (service users) related to their family relationships, biological, or genetics dispositions, critical events, interpersonal interactions, and economic and social environments. The movement toward empathy and compassion is foundational to the integration of empowerment strategies into practice. Consideration of the external contextual factors of mental health is a key starting point for the intersectional empowerment process (61).

*“In talking to the student, I would also learn about him. He would talk about why he has psychological problems, and I would find out that his emotional outlooks and some of values were greatly shaped by his family of origin and have an inextricable relationship with his parents’ influence on him.” (FG1-2)*

Second, empowerment took place when the participants began to recognize the strengths of the service users themselves. A philosophy of service enhancement emerged among some MHSP participants, who began to focus on the students’ personal strengths, rights, and competencies rather than their deficiencies. They expressed a commitment to creating a therapeutic environment in which the student service users feel respected, supported, and valued.

*“We were praising you (referring to a student who was suffering from major depression and wanted to drop out) from the bottom of our hearts, (telling the student) ‘That’s how you are, that’s just great,’ and by encouraging him and then letting him embrace himself, he’s getting better now, too.” (FG3-5)*

These service providers expressed a preference for choosing to believe in students and conveying their confidence in them in their services. They were able to provide a sense of hope and motivation among students with psychological problems, empowering them to self-manage and connecting them to support networks, thus improving their self-preservation during illness and life, and family–social connections (62, 63).

*“We spread the new knowledge among the students and tell them how important it is. For the faculty and staff, this is another aspect of their employment, as it is for everyone else who spreads the word. Now, because of the establishment of a parents’ contact (network communication) group (on WeChat), this role will also be given to the parents. They will be told to pay attention to the students’ mental health. So, some of these support roles are also established for the parents, and in this way, all-round psychological promotion (care) for the students is provided.” (FG3-1)*

In terms of empowerment actions, the MHSPs identified more cooperation, encouragement of participation, and ceding of decision-making power as key processes and outcomes. Some MHSPs expressed greater willingness to provide emotional and informational support and to share their experiences in implementing helping services when responding to university students’ mental health issues. In addition, many of the MHSPs mentioned that interactions on topics concerning mental health focus on guiding student participation and building partnerships with university students. In healthcare, empowerment interventions are a collaborative approach centered on the service user, resulting in a complex experience of personal change facilitated by healthcare workers (64). The participation of service users was considered an essential prerequisite for empowerment (65) because of the empowerment effect of engagement and the mutual mobilization of each other’s capacity. The MHSPs increased their synergistic interactions with service users (university students), prioritized their engagement in personal mental health promotion, and school mental health culture and encouraged the university students to build on their strengths and engage in collective action for health improvement. This situation suggests that service providers are particularly inclined to view empowerment in terms of increasing individuals’ knowledge, skills, and self-confidence (66).

*“There are a series of knowledge science, lectures, group counseling, and some fun mental health education activities on my (university’s) side, and through a series of such actions, students can participate in a wide range of activities to achieve holistic mental health education and promotion and the reduce stigmatized perceptions (of mental illness).” (FG3-2)*

### 3.2 Theme 2: Professional insights and motivation for organizational empowerment

Mental health researchers have argued that empowerment in recovery discourse is primarily focused on the individual level, meaning that interpretations or interventions on the wider societal level are increasingly suppressed or depoliticized in the implementation of recovery approaches (67). In contrast, in the MHSPs focus group interviews for this study, it was found that by engaging in empowerment training, it was possible to increase professional insight into organizational empowerment and advocate for action at a wider level to reduce the stigma associated with psychological problems, and call for more policy adjustments.

Initially, it is important to recognize the structural and systemic barriers to mental health. Through the discovery of factors outside of the individual service users’ system, a dialogue to explore organizational dilemmas, policy imperfections, and environmental impediments can begin. This, in turn, leads to a collective sense of empowerment and intention. True empowerment is a process that can be facilitated by removing barriers (68). Insights for collective empowerment are stimulated through collaborative group learning. These new insights empower the MHSPs to become practitioners engaging in (initiating) change. This leads to the development of safe contexts characterized by trust and collaborative partnerships that promote empowerment.

*“The bigger obstacle is just the whole climate, I guess. This year is actually a little bit better; perhaps the teachers have a better feel for (i.e., understanding of) mental health, but in previous years it was not as good. Although we have been concerned about mental health issues, from the policy perspective to implementation at the school level, we still have limited knowledge. There is little we can do in that regard.” (FG3-2)*

Furthermore, it is vital to move toward improving the mental health environment. Our interview data revealed that service providers strive to get involved in efforts to build interdependent and caring environments. MHSPs (both professional and non-professional) are called upon and encouraged to participate in group empowerment initiatives (69). After the ACE-LYNX training, the service providers conducted counseling, education, and advocacy activities, such as parent psychoeducation seminars and mental health promotion months, targeting the external environments affecting mental health and illness recovery, such as families, dormitories, classes, and communities. The aim was to shape interdependent and supportive external environments conducive to psychological interventions and illness recovery.

*“Our school has been establishing initiatives to single out mental health as one of the issues about which people should be better informed. Advice has been given to students, staff, and parents with the aim of spreading knowledge regarding mental health. The goal is to help them to understand each other, help each other when there are difficulties, and ensure that they understand the importance of this message. The information is also circulated in the parents’ (network communication) group. I think our school will do a better job this year and our university will also do a good job.”(FG3-1)*

Meanwhile, it is essential to advocate for the promotion of multi-actor collaboration. The service providers in the focus groups expressed their expectations regarding the multifaceted implementation of mental health facilitating actions at the student, college, school, inter-school, and school-hospital levels. Empowerment is both a concept that brings about desired outcomes and a process of social action. Having broader institutional values and policies helps create campus environments (70) that value student mental health and enhance supportive and inclusive service practices.

*“This implementation also requires some cooperation between different parts of the school and the school and the community; for example, the school and the hospital. Support is required from the top to the bottom, and is also given to us, and we look for some cooperation and assistance from the school itself.” (FG1-6)*

### 3.3 Theme 3: Non-self in the continuum of collective empowerment

A key empowerment concept in ACE-LYNX is “interdependence”. Through experiential learning activities, many participants recognized that the “self” does not exist independently of other people and the environment in which they live. This insight motivated them to move beyond individual service provision to become mental health champions, thus moving along the empowerment continuum to take action in groups and communities.

*“Through this kind of activity, I feel like I’ve become more flexible—that is, first of all, there is a certain epiphany from my own life, there is a certain sublimation, so that the quality of my life has become improved. I give classes to students, and I’m trying to promote this part of the program and also trying to make a link between everyone, every person, the interdependence between people, and their connection to the world. By promoting the program and spreading it, it will help more people.” (FG2-3)*

Many of the participants shared that the ACE model’s integrated empowerment pedagogy enabled them to develop psychological flexibility, which propelled them along the empowerment continuum with sustained momentum. Many became mental health champions beyond their workplaces.

First, experiential learning with interactive games helped contextualize and concretize the concept of empowerment. Some service providers indicated that they were relatively unfamiliar with the concept of empowerment and that they felt that its contents were implicit. The ACE-LYNX training was based on various scenario-based experiential game activities that were used to guide service providers to better understand the knowledge and practice of empowerment. Despite being novices in practicing these ACE activities, many of the participants indicated that they remembered the game interactions well and that the impact of the activity experiences helped them understand empowerment.

*“The thing that really struck me was the Exclusion Circle. I was really rejecting these kinds of students (students with psychological problems) before—I had this kind of emotion. But after I participated in (the ACE-LYNX) intervention, I thought that (for them) this (psychological problem) is an object (that is) very impersonal, very normal, so since then, when I face any kind of (difficult situation), especially (with) students,……I think it’s very normal when they have abnormal emotions, and I think it’s very normal, and I think it’s very clinical” (FG1-3)*

Empathy is the best accelerator in the progression from individual empowerment to higher levels of organizational and community empowerment. The MHSPs placed a greater emphasis on empathetic narratives (71), such as openly listening to the experiences and feelings of others, engaging in self-disclosure, respecting when and what students with psychological problems wished to share, as well as considering their unique insights, strengths, and experiences.

*“I think the first thing to do for the students (service) is to be empathetic—even though I may not be able to empathize with them, I will listen to them (the students) and try to empathize with them as much as I can. I think that this is the way to gain the students’ trust, and then the effect of the heart-to-heart talk is also better” (FG1-2)*

In listening to the service recipient’s story, the service provider facilitated his or her own understanding of the situation and circumstances, thereby adding to and creating meaning connected to the caseworker’s experience. The MHSPs reported that they tried to demonstrate attitudes that contributed to the empowerment process in follow-up services, such as engaging in self-reflective conversations and attending to the patient’s priorities and concerns. The process of empathizing with the service users involves the service provider realizing that in addition to providing support, they should perceive and take action in terms of the larger system.

*“I tried to find a way to understand him and then switched to his perspective to experience his feelings. By doing so, I actually helped a few students when dealing with their academic work. Because some of the students’ situations are really tricky, there are times when the teachers really can’t take it in, and then I (when I encountered a similar situation again) felt that (he) was normal, and that we could understand him, (because) he himself is suffering first of all.” (FG1-3)*

The MHSPs claimed to have achieved full-level empowerment through the experience–reflection–action cycle. Experiential learning is a key feature of group empowerment education. Group discussions, practical exercises and self-reflection are organized after explanations related to empowerment topics so that participating service users can set individual goals for themselves and plan how to practice at home. The providers can use what they have learned to reflect on their current practices through a mental health lens to support student well-being and evaluate how they might be supporting or hindering students. The project curriculum encourages participants to think beyond their own efforts and those of their organizations and to begin to shift their perspective to the broader impacts, with a deeper understanding of social justice and other wider meanings.

*“If you go back to the whole social and community level, what kind of attitude did I have toward them? It caused some reflection and growth in myself.” (FG1-8)*

### 3.4 Theme 4: Interdisciplinary challenges and divergences in empowerment action

Effective practice in school mental health is highly dependent on cross-disciplinary cooperation (72). The MHSPs in this study had a variety of professional backgrounds, including psychiatry, nursing, social work, and psychology. The advisors had more complex educational backgrounds (including polytechnic education and management sciences qualifications). MHSPs from different disciplines face different constraints and challenges in their service provision (73, 74). They might engage with empowerment differently and present divergences in action. At the same time, the shared mental health championship in the Linking Hearts Project also seemed to unify them in committed action.

In this study, the psychiatric service providers suggested that the empowerment orientation in ACE-LYNX had prepared them to add new dimensions to their services in a clinical setting. However, they highlighted the barriers and challenges involved. In China, by the time individuals are hospitalized, they are often experiencing severe illness symptoms of illness (75), making it difficult to engage them in ACE activities until they are stabilized. In addition, social stigma interferes with the understanding by family members, who are already filled with anxiety about their loved ones’ illness (76). All of these pose challenges in therapeutic interactions. As FG1-1 described that:

*“In the past, when we talked to a patient about his/her medical history, we (only) looked at the records. After the activity, when I talk to the patients alone, we (will) listen to the patient himself, ask him(the patient) which point he doesn’t understand, or ask the patient directly—I personally (try to) get to have an understanding of the patient, and (this happens) quite a bit.”(FG1-1)*

*“After all, there is still a gap between our work (hospital psychotherapy work) and the work of university teachers (university counselors and the staff at psychological centers). Then again, we are working in the hospital. In the wards it is difficult to carry out these kinds of activities (experiential reflective activities). After all, they are already severe psychiatric patients. In addition (regarding mental illness), there is stigmatization. After we talk about it with them (the patient’s family), the family may inquire about how and why he (the patient) is suffering from this kind of disease. They (the family) may focus on the idea of genetic aspects, which may be considered embarrassing” (FG1 -1).*

Unlike the psychiatrists, many university counselors were very appreciative of empowerment practices and had a deeper understanding of the individual-level values and knowledge of respect, skill development, self-worth, competence, and self-efficacy advocated by empowerment-oriented practices. This subsequently increased the MHSPs’ inclusion and willingness to implement counseling practices for students. However, some non-specialized MHSPs also expressed concerns about the practical application of empowerment practices for use at the community level.

*“In the past, I knew that depression is a mental illness that needs to be treated, but I was not so tolerant of him (referring to a student suffering from major depression) and his behavior. After learning this, my tolerance for him has increased. Maybe it’s the “Compassion Meditation” (a practice activity), which (encourages) a kind of compassion, leading to acceptance that he is really in this predicament, and empathizing with his predicament, and transmitting this compassion to him. We’re learning these tools, and the experience is good. I just don’t know exactly how to use them yet, and I don’t know what support I need, what I can use.” (FG2-5)*

In contrast, MHSPs with a professional background in social work have come to the fore in the translation of empowerment practices, and their actions have gone beyond frontline support and community advocacy for service users. This has enabled reflection on organizational policy reform and the cultivation of a social and cultural climate for mental health. This is because social work values, particularly the promotion of self-determination, strength-based perspectives, and social justice, are seen to be very much aligned with the underlying principles of recent mental health initiatives, including recovery-oriented, individualized service models (77).

*“At the student level, by giving them meetings or having class meetings and so on, this knowledge has been popularized. There is a realization of the need for social justice, fairness, and equality—that in the whole society, in the whole collective, we need to be interdependent. We promote these concepts and then let them slowly infect every different student.” (FG3-4)*

In the ACE-LYNX intervention, MHSPs from different disciplines were brought together to learn, collaborate, and work as champions to reduce the stigma around mental illness and promote mental health among students. In this context, despite the differences in their disciplinary perspectives, their shared goals and collaboration became potential resources that could enrich the pathway to collective empowerment.

## 4 Discussion

The importance of empowerment is emphasized in research on and the practice of mental health recovery (15, 17, 18). The empowerment of MHSPs is consistent with the goal of recovery-oriented services that advocate for the engagement of service users. Previous research has suggested that MHSPs lack the necessary knowledge, clarity, and experience to apply empowerment principles and that their ability to adapt empowerment practices to serve the needs of diverse service users therefore remains inadequate (78). In this regard, empowerment education and training mental health champions have become important mechanisms for improving skills and facilitating empowerment actions (79). This study analyzed the performance of service providers’ empowerment interventions with the aim of providing applicable experiences to promote empowerment practices among MHSPs.

Regarding the effectiveness of ACE-LYNX in promoting empowerment practices, we found that the intervention not only increased the MHSPs’ conscious awareness and brought about behavioral change through psychological empowerment, but also stimulated their professional insights into and motivation for organizational empowerment. This concurs with the findings of previous research on the pathways of empowerment education to realize the impact of the transfer of awareness, following the awareness-to-adherence model in the Healthcare Practitioner’s Guide (80). In group empowerment education, the process of uptake of empowerment by MHSPs passes through sequential, cognitive, and behavioral steps. First, there is awareness of empowerment, then intellectual endorsement of empowerment, followed by a decision to adopt empowerment in practice, and finally, in due course, actual successful adherence to empowerment practices. This again confirms that taking up the role of a health champion can increase the success of the timely integration of empowerment strategies into current practice and ultimately improve the delivery of mental health services (81).

We also found that the experience of empowerment at the individual empowerment level can be profound, immediate, and transformative. Many participants spoke passionately about their liberating experience of psychological empowerment, including increased confidence or improved self-concept as a result of collaborative learning or action (82). In the contexts of MHSPs, intrapersonal empowerment occurred through the practice of defusion from socialized self-blame, stigma, misconceptions, and prejudice toward vulnerable groups. This empowerment became interactional when the MHSPs integrated empathy, compassion, and advocacy into their practice, demonstrating humility, deep listening, respect, and high regards toward the service users as they provided support in problem solving, emotional support, and skill learning opportunities. Furthermore, as some participants demonstrated, once they reached psychological empowerment, they were able to view student mental health care in the context of organizational structures and policies. While this study did not capture the outcomes of organizational empowerment, which requires a longer time period, we were able to show that individual empowerment is an essential step toward and driving force in promoting organizational empowerment.

Conversely, at the community and societal levels, transformative activities are fostered through the joint participation of many stakeholders (decision-makers, community leaders, service providers, service users, etc.) and within the constraints of societal structures and policies. Community empowerment involves complex and often difficult processes. Due to the collective nature of community empowerment, it is often difficult for individuals to feel the experience of empowerment directly (83). As indicated in previous studies, community empowerment is an interplay between individual and community change over a long time-frame (84). However, psychological empowerment is a construct that incorporates the person’s perceptions and actions within their social context (85). Through interdependent interactions, individuals work toward the ultimate goal of collective empowerment (social and policy change) (86).

Our study’s results are consistent with previous findings on the multi-level nature of the concept of empowerment (87) and the dynamic nature of the empowerment process (88). We found that the empowerment practice of MHSPs expanded from the individual to the societal level through the links created by knowledge combination and the catalytic role of compassion in the ACE model. Compassion enabled the MHSPs to empathize with the service users’ psychological challenges to believe in the interdependence of members of the community, and to engage consciously in collective empowerment actions that promote social change. The learning process in the ACE-LYNX intervention was based on interactive simulation games with metaphorical meanings that enabled participants to visualize the implicit conceptual connotations. The group sharing also provided opportunities to build “social relationships” that were critical to moving participants toward their shared values, common goals, and finally committed action.

Additionally, the integration of empowerment into practices requires continuous engagement in the experience–action–reflection cycle. Previous studies have highlighted the contribution of competency-based approaches to the theory of change. Such approaches can guide participation and facilitate empowerment, and the concept of critical pedagogy is an important component of the ACE-LYNX intervention and other empowerment learning systems (89). Comprehensive interactive training strategies (experiential self-exploration, group interactive exercises and reflection) in group-based empowerment psychoeducation facilitate the active participation of MHSPs. As demonstrated in the ACE-LYNX intervention, empowerment occurs through an ongoing relationship of participatory dialogue, reciprocity, and equality that promotes self-reflection, understanding, and motivation to contribute to their organizations and communities. Participatory empowerment education is rooted in critical consciousness theory and promotes power sharing and the understanding of lived experiences. It also encourages participants to construct and share their reality and to take collective action for positive change. Through a continuous cycle of iterative critical reflection and dialogue, MHSPs are prompted to become aware of the structural and systemic limitations of psychological problems, allowing them to recognize that the plight of people with psychological problems requires not only solutions at the individual level, but also connections to the broader societal context and sensitivity to structural issues such as social injustice, which activates agendas around social justice.

This study found that there are diverse perspectives and divergences in empowerment action among MHSPs. This is consistent with the findings of previous studies on the understanding of multidisciplinary dynamics in promoting empowerment in mental health practice (90). This may be related to the fact that empowerment itself is a multifaceted concept (87), as well as to the disciplinary boundaries and cultures of the values of different professions, as revealed by studies of multidisciplinary collaborative empowerment. MHSPs from social work backgrounds continued to emphasize the community/social action dimension of empowerment practice: professionals from psychiatry backgrounds focused more on personal empowerment. There are also different degrees of knowledge–doing gaps among service providers of different specializations in the team due to differences in their understanding of empowerment and paradigms of action. The knowledge–doing gap is the difference between what health service providers know through education and experience and what they actually do when working with “real” users (91). This could be studied in more depth by researchers in the future, as the results of the knowledge-action gap analysis could help policymakers understand whether interventions are likely to be an effective means of improving health services, as well as identifying poor overall quality in providing mental health care and thus the root causes. The policy system for responding to mental health problems requires multidisciplinary decision-making to safeguard the well-being and rights of service users (92, 93).

Our study also has some limitations to our study. The first concerns the selection of the participants, who were mostly self-enrolled and were predominantly advisors. They were, therefore, part-time MHSPs in higher education for whom mental health services are not their primary responsibility. Second, as mentioned previously, this paper reports on one of many topics in the ACE-LYNX intervention, and uses mixed methods for evaluation, including quantitative data on MHSPs’ attitudes toward stigma and psychological resilience. A paper to report on the findings of our mixed-methods research and data triangulation could further contribute to knowledge on empowerment outcomes. Additionally, this is a cross-cultural implementation study. The ACE model was implemented and evaluated in Asian, Black, Latinx and other racialized communities in Canada. The influence of sociocultural and contextual factors cannot be ignored, especially in the conceptualization and understanding of multi-level empowerment. Discussion of these complexities was beyond the limits of this paper, but our team is analyzing this for future research and knowledge dissemination. Despite these limitations, this study provides evidence of the importance and potential benefits of integrating empowerment into the training and professional development of MHSPs and engaging them in becoming mental health champions beyond their workplace settings. Furthermore, this paper shows that after receiving empowerment education, MHSPs can play an important role in conducting collective mental health empowerment to reduce the stigma associated with mental illness.

## 5 Conclusion

Empowerment is recognized as a critical component of mental health care. The integration of empowerment education into professional training is critical. As illustrated in this paper, the process of developing an empowerment practice is characterized by the MHSPs moving from individual to collective empowerment along a continuum. As empowered MHSPs interact with each other and collaborate in working toward their shared goals, they contribute to organizational empowerment, which will, over time, advance community empowerment. Experiential learning, empathy education, and critical reflection can accelerate the continuous iterative transformative process of empowerment practices. To advance the integration of empowerment in mental health care, the engagement of organizational decision-makers and policymakers in empowerment training is also critical to ensure the alignment of empowerment values and competence at all levels of service provision. Building empowerment competence among decision-makers will contribute to more effective and inclusive policy decisions and equitable distribution of resources. Further research is needed to explore cross-cultural adaptation and implementation of empowerment education to advance knowledge and practice.

## Data availability statement

The datasets generated and analyzed during the current study are not publicly available due to restrictions placed on data access by the Research Ethics Boards of Shandong University, Toronto Metropolitan University and University of Toronto, and other affiliated universities, but are available from the corresponding author on reasonable request.

## Ethics statement

The studies involving humans were approved by Toronto Metropolitan University (formerly Ryerson University), University of British Columbia Okanagan, University of Toronto, York University, University of Alberta, Shandong University, Shandong Jianzhu University, Jinan University, Shandong Mental Health Center, Shandong Normal University, Shandong Women’s University, Shandong Youth University of Political Sciences. The studies were conducted in accordance with the local legislation and institutional requirements. The participants provided their written informed consent to participate in this study.

## Author contributions

FW: Data curation, Formal analysis, Visualization, Writing – original draft, Writing – review & editing. JG: Conceptualization, Methodology, Supervision, Writing – review & editing. SH: Formal analysis, Writing – review & editing. KT: Methodology, Validation, Writing – review & editing. JP-HW: Conceptualization, Methodology, Supervision, Validation, Writing – review & editing. KF: Methodology, Project administration, Resources, Supervision, Validation, Writing – review & editing. AT-WL: Conceptualization, Project administration, Supervision, Writing – review & editing. CJ: Funding acquisition, Investigation, Writing – review & editing. SC: Data curation, Investigation, Project administration, Writing – review & editing.

## Footnotes

1 They, called Fu DaoYuan in Chinese, are personnel engaged in the daily ideological and political education of university students, and mental health education and psychological counselling is one of their important duties. There are full-time and part-time advisors, of which the full-time advisors include the deputy secretaries of the Party committees (party branches) of the school (departments), the heads of the academic and labor groups, and the secretaries of the Youth League committees (Youth League branches), etc.; the part-time advisors are selected and recruited from among the outstanding full-time teachers, administrators, and postgraduates.

2 Each university has a Student Mental Health Education and Counselling Center, staffed by professional psychological counsellors [Each university will be staffed at a faculty-to-student ratio of not less than 1:4,000 and at least two per university.], which undertakes mental health education courses and psychological services for all students. It also provides training in psychological helping skills for full-time and part-time advisors, teachers, and student peer counsellors.

3 The Ministry of Education advocates for the development of a sound collaborative mechanism between schools and mental health medical institutions to jointly deliver mental health services to students.
